# The Milk of Eclamptic Mothers

**Published:** 1912-03-09

**Authors:** 


### THE MILK OF ECLAMPTIC MOTHERS.

### Its Toxicity to Infants Considered.

Among the problems of obstetric medicine there are few which are so puzzling and so many-sided as those connected with the causation, pathology, and treatment of eclampsia. On nearly every point connected with this obscure condition opinions vary widely; probably because no line of treatment hitherto followed has succeeded in reducing the terrors of puerperal convulsions to vanishing point or anywhere near it. It is, of course, pretty generally agreed that a maternal toxæmia of some kind or another—fœtal, autogenous, nephritic, or what not—underlies the manifestations of the disease; and much research has been done upon the toxic constituents of the blood serum, cerebro-spinal fluid, and urine of those in whom eclampsia has occurred. Strangely enough, the toxicity of the milk seems to have attracted hitherto no attention whatever; at least no publicity has been afforded to any facts or reflections on the subject.

### An Important Question.

Unusual interest attaches, therefore, to a very able paper * in which Dr. J. R. Goodall propounds the query, Should eclamptic mothers nurse their new-born? This question, considered from the point of view of the interests of the child, is quite a new one. As bearing upon it, the author quotes three cases, which have come under his own observation, of sudden death without apparent cause in seemingly healthy infants after their first copious nursing; in each instance the mother had exhibited signs of eclampsia. The children died so suddenly and with such a similar symptom complex that he is compelled to think that their exitus was from a common cause. Without laying down any dogmatic doctrine, he deals with the subject on broad lines, hoping to elicit further evidence bearing on the problem.

The first case was that of healthy multipara, an exceptionally fine specimen of womanhood and motherhood, who was delivered at term of her fourth child, a strong healthy infant which cried lustily immediately after birth. The labour was easy and natural; no signs of eclampsia were noticed until two days after delivery, when the patient was suddenly convulsed and became comatose two hours after nursing her child, which had then for the first time obtained a copious supply of milk. In twelve hours she was dead; but meanwhile, five hours after the aforesaid nursing, the baby was seen to be cyanosed and cold. Somnolence, twitchings, and rigidity of the extremities rapidly developed with abdominal distension and Cheyne-Stokes respiration. The child died a few hours before the mother.

The next case shows many points of similarity in outline, though differing in details. A woman of twenty-eight had albuminuria and casts for the last month of pregnancy; and for the last week before delivery complained of severe headache and abdominal pain. Labour was easy, but the headache still continued, and thirty-two hours afterwards she became definitely eclamptic. After thirty fits, spread over the next day and a half, she recovered. The child was put regularly to the breasts, but got little food and cried. On the third day it got satisfaction for the first time and ceased to cry. Six hours later it was cold and cyanosed. Respiration became slow and shallow, and there were muscular twitchings with occasional nystagmus. Somnolence and cyanosis deepened, distension developed, and death speedily ensued.

In the third patient hydramnios and accentuated aortic second sound were discovered at the seventh month. Suspicion thus aroused led to the discovery of marked albuminuria. Under treatment (rest, milk diet) she improved; and the albumin diminished temporarily, but rose again before labour. There was neither headache nor vomiting. Labour was quite normal, except for a few twitchings of the face in the second stage. On the second day after labour there were severe headache and loss of appetite, though the bowels had moved freely. Albumin was still present in the urine, with casts and bile; there was also slight jaundice. Next day she seemed better, and remarked that the milk had flowed freely for the first time that morning. She also alluded with gratification to the improvement in the baby's behaviour in the matter of noise. On inspection of the child the same clinical picture presented itself: cyanosis, cold extremities, muscular rigidity, and shallow irregular respiration. The child died the same night; the mother had no fits, but it was six months before the urine was free from albumin.

The consideration of these extraordinarily interesting cases leads to several queries. Was there any connection, and if so, what, between the nursing and the incidence of the sudden change in the child's health? What was the nature of the disease which caused the death of these three babies? Is the milk of an eclamptic mother toxic? And if so, why should it be toxic to the child which has been nourished on her own blood? Are the children of eclamptic mothers healthy? Should they be nursed or artificially fed?

### Some Theories.

After careful search of the literature Dr. Goodall has found but one analogous case; but he quotes many authorities on some of the questions propounded. Many cases have been published of convulsions in the new-born infants of eclamptic mothers, and autopsies on fatal cases have shown kidney changes and other lesions similar to those of eclamptic mothers. Convulsions *in utero* have also been described and are generally believed to be possible occurrences. Further, he believes that eclampsia in the mother may be a source of chronic nephritis in young children, and that this condition underlies infantile convulsions in a much larger proportion of cases than is generally acknowledged.

That the milk of an eclamptic mother should be

* *American Journal of Obstetrics*, January 1911, p. 11.

toxic would seem not unnatural. Even bacteria can pass from the circulation into the milk, and therefore it would be strange if soluble toxins are unable to do the same. Still, if an infant can thrive on its mother's toxic blood right up to full term, and be born lusty and healthy, it is certainly difficult to comprehend why it should take any harm from milk manufactured direct from the same source—the maternal blood-stream. To meet this objection the author puts forward the hypothetical possibility that the milk may secrete a concentrated accumulative toxin more virulent than that which circulates in the blood. In support of this he points out that mercury exhibited to a nursing mother has more than once caused alarming symptoms of mercurial poisoning in the child. On one occasion the author had to treat gallstone colic during labour; he gave 2.25 grains of morphia in twenty minutes, without the slightest ill effect on the child as far as could be ascertained. Yet six days later a quarter of a grain of the same drug led to most serious symptoms in the nursling, which got, he supposes, a larger or more concentrated dose through the milk than it did through the placental circulation. Furthermore, a case has been reported in which the administration of arsenic to a mother caused severe diarrhœa in the child. On estimating the excretion of the drug, it was found that one part of milk contained as much arsenic as ten parts of urine. As soon as the arsenic treatment was stopped the child ceased to have diarrhœa.

The difficulty which has to be got over before this assumption can be entertained is that in the majority of cases the nurslings of eclamptics exhibit no signs of toxæmia at all. To meet such an objection Dr. Goodall points out that these particular cases differed from the ordinary run in that the markedly eclamptic symptoms did not come on before or immediately after delivery, but two or three days after. In ordinary cases delivery is followed by tremendous diuresis with diminution or cessation of fits; in other words, a great quantity of toxin is eliminated while the breasts are almost inactive, and before the milk secretion is at all free. So that when this latter function becomes well established there is no longer any large amount of toxin to eliminate. But when, owing to some grave antecedent chronic renal lesion or other causes not yet fathomed, the toxæmia reaches its height coincidently with the first copious lactation, then the milk may quite plausibly be expected to set up grave symptoms in the infant, as in the three cases narrated.

Such are the highly ingenious and interesting speculations of the author as to the explanation of the three sudden infantile deaths. He concludes with some recommendations based upon the cases. With a mother markedly toxæmic, especially if jaundiced, he would feed the infant artificially for a few days, pumping the breasts dry once or twice after the toxæmia improves before the child is put to them. If convulsions come on *post-partum*, allow toxin elimination to go on until the symptoms have practically subsided before putting the child to the breast; and, as before, use the pump to empty the breasts before allowing the mother to suckle. If albuminuria persists after gestation, feed the child artificially throughout.

